# Negative Pressure Wound Therapy Can Prevent Surgical Site Infections Following Sternal and Rib Fixation in Trauma Patients: Experience From a Single-Institution Cohort Study

**DOI:** 10.7759/cureus.9389

**Published:** 2020-07-25

**Authors:** Daud Lodin, Taylor Florio, Thomas Genuit, Nir Hus

**Affiliations:** 1 Surgery, Florida Atlantic University Charles E. Schmidt College of Medicine, Boca Raton, USA; 2 Surgery, St. George's University School of Medicine, St. George's, GRD; 3 Surgery, Delray Medical Center, Delray Beach, USA

**Keywords:** chest wall reconstruction, wound vacuum, thoracic surgery, reconstruction, trauma, sternal fixation, rib fixation, surgery, negative pressure dressing, closed-incision negative pressure therapy

## Abstract

The management of patients with traumatic injuries can be a challenge. Many require surgical intervention, are at an increased risk of surgical site infections (SSIs), and have an associated increase in hospital length of stay and cost. Closed-incision negative pressure therapy (ciNPT) has shown benefits in the management of certain surgical sites by preventing infection and improving wound healing. In the setting of chest wall reconstruction after traumatic sternal and/or rib fractures, no study so far has examined the efficacy of this treatment. We report a single-center retrospective cohort study, examining outcomes using ciNPT following rib and sternal fixation in trauma patients. Data on 71 patients who suffered from rib and/or sternal fractures, requiring surgical intervention, were collected over a time period of three years, from December 2016 to December 2019. The patient population was 66% male (47/71), had a mean age of 63.3 years (range 23-90 years old), and suffered from injuries related to motor vehicle or motorcycle accidents (45/71, 63%). Among the patients treated with ciNPT, none developed signs of SSIs during their initial hospitalization or within two months post-discharge follow-up. Negative pressure therapy is an effective wound care management system for preventing infections in closed-incision sites following chest wall reconstruction.

## Introduction

Approximately 290,000 cases of surgical site infections (SSIs) occur every year in the United States and add an estimated $3.5 to $10 billion to healthcare costs annually [1]. Many of these infections occur in trauma patients and can be attributed to traumatic wound contamination, transient tissue hypoperfusion from sepsis and shock, altered patient immune response, and the need for multiple procedures [2]. Wound infection after sternotomy occurs in 1%-5% and after surgical intervention for rib fractures in 2%-7% (range 0.5%-20%) of patients [3,4].

Closed-incision negative pressure therapy (ciNPT) was first developed as a strategy to reduce peri-operative SSIs in the 1980s and modified for commercial use in the 1990s [5]. ciNPT applies continuous sub-atmospheric pressure to surgical incisions, reducing wound edema, bacterial load, and seroma formation [5]. Additional proposed mechanisms aiding in the reduction of SSIs include prevention of adhesion, inhibition of pathogenic organisms, and promotion of blood flow [5]. Currently, ciNPT is well established in the management of open wounds in trauma and other surgical patients. It is utilized in patients who have undergone laparotomy, orthopedic procedures, amputations, and skin grafting. In cardiac surgery patients, ciNPT is regularly used to manage sternal infections [5].

The surgical stabilization of rib and sternal fractures has become a common treatment modality in trauma patients to address the sequelae of chest wall instability (flail chest) and help with pain management [6]. Although usually clean wounds, with the exception of open fractures, patients undergoing these procedures may be increasingly prone to SSI [7]. SSI after rib fixation carries the increased risk of hardware infection and osteomyelitis, which may require hardware removal, prolonged wound management, and antibiotic therapy. Proximity to penetrating wounds or evidence of systemic infection significantly increase the risk of SSI after chest wall reconstruction [6].

To date, there is no peer-reviewed publication to assess the use of ciNPT after sternal and/or rib fixation in trauma patients. This study reports on a single-center cohort of 71 patients who suffered from sternal and/or rib fractures, requiring surgical fixation and treatment with ciNPT.

## Materials and methods

**Figure 1:**
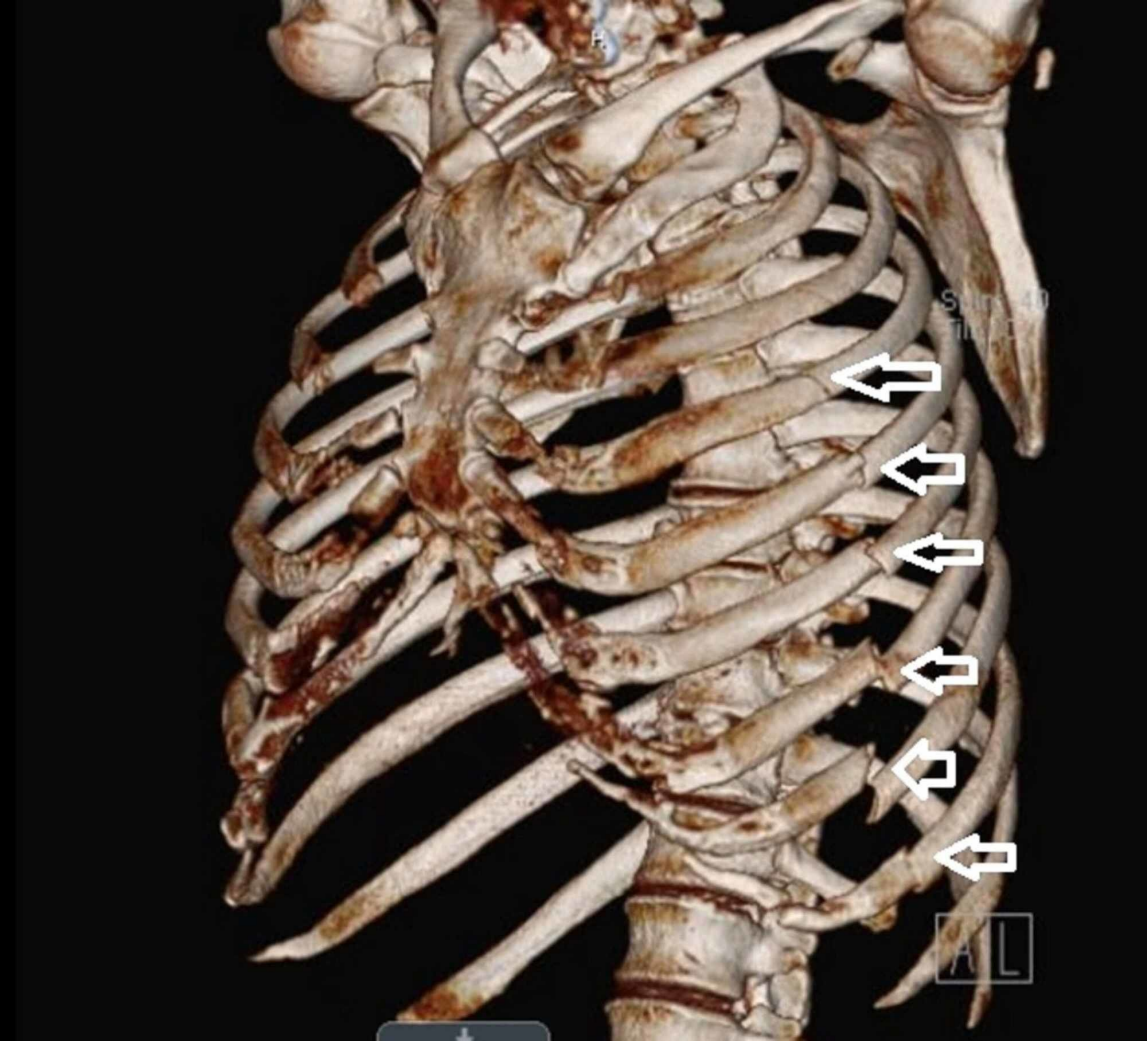
Example of chest wall 3D reconstruction via computer tomography 3D, three-dimensional. The white arrows indicate the left-sided rib fractures of the fourth, fifth, sixth, seventh, eighth, and ninth ribs, with mild displacement of the sixth, seventh, eighth and ninth rib fractures.

**Figure 2:**
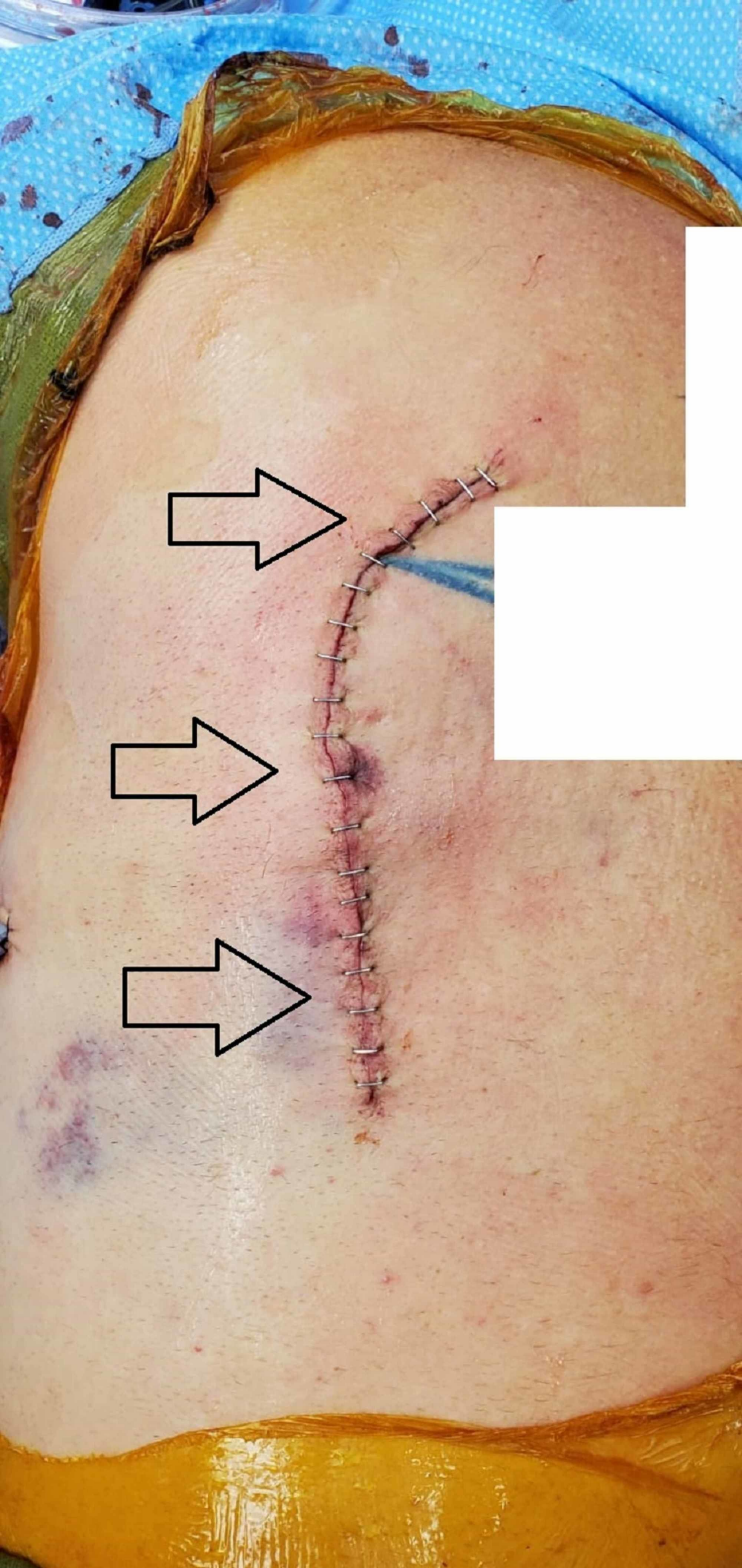
Right-sided chest incision after rib fixation The arrows indicate the location of the incision (care was taken to remove any identifying tattoos in the image).

**Figure 3:**
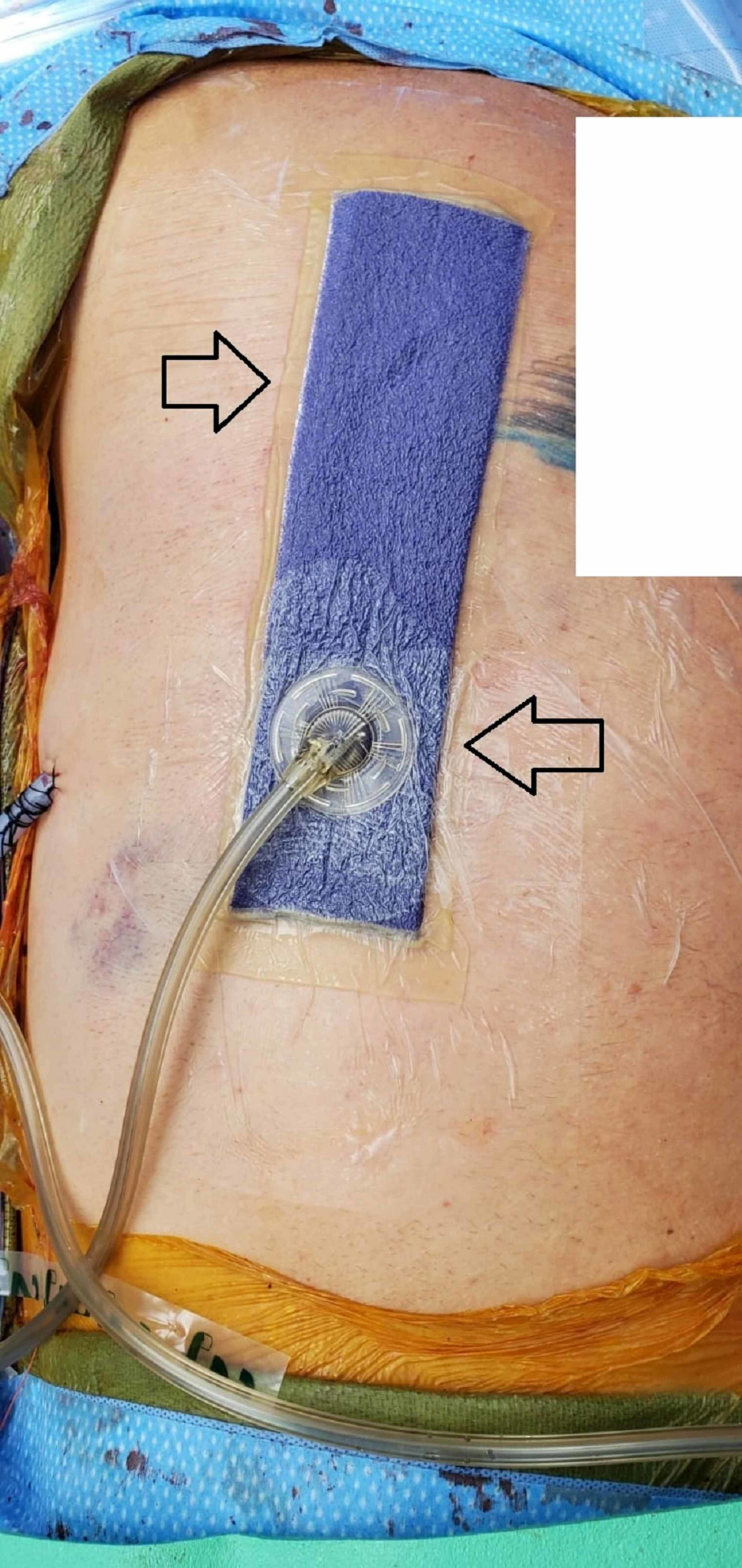
Right chest placement of the PREVENA™ wound management system over the right-sided chest incision This image is from post-operative day 7 prior to removal. The arrows indicate the location of the wound sponge under suction.

**Figure 4:**
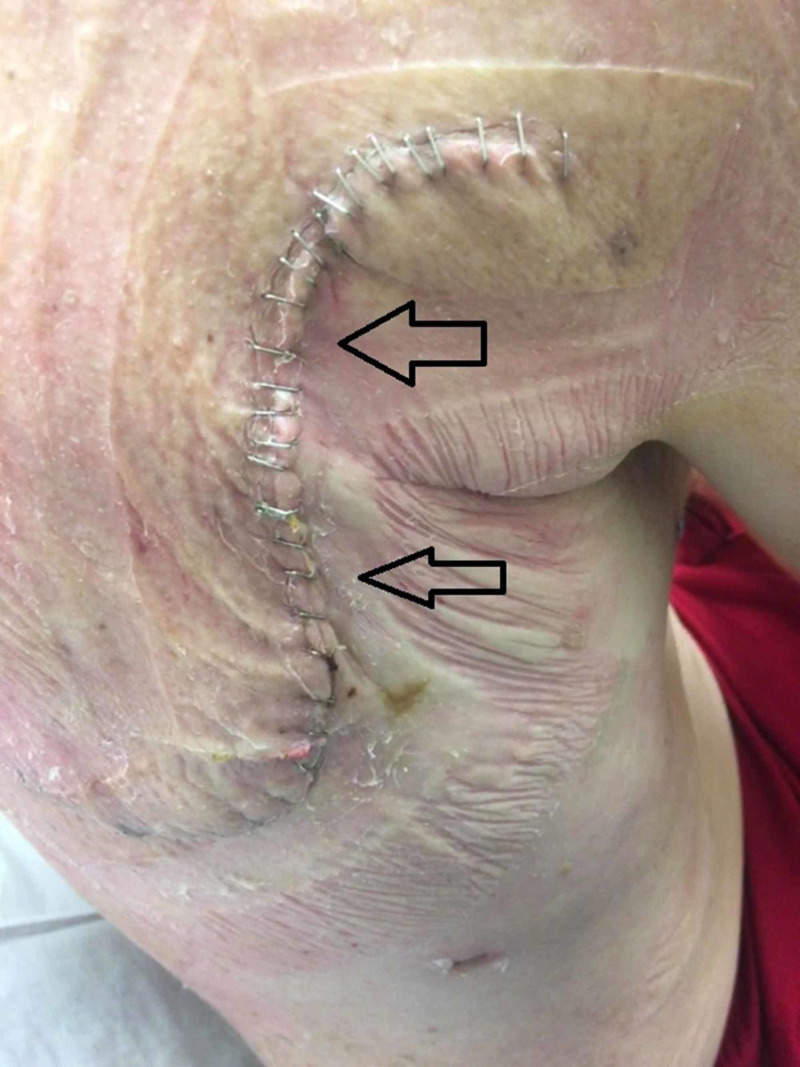
Right-sided chest incision site after the removal of the PREVENA™ wound management system The arrows indicate the incision site with staples used for closure. Notice that there is no significant edema, erythema, or wound dehiscence on post-operative day 7.

Electronic patient medical records at a Level I trauma center were searched for patients with current procedural terminology codes 97606 (placement of a negative pressure dressing system) and 32820 (major chest wall reconstruction following thoracic trauma) over a period of three years (December 2016 to December 2019). Inclusion criteria included adult trauma patients (age 18-90 years) with traumatic rib or sternal fractures, who underwent fixation with placement of a closed-incision negative pressure dressing immediately post-operatively. Seven patients with incomplete data were excluded from the analysis. As part of their work-up and pre-operative planning, all patients underwent a computer tomography scan of the chest with three-dimensional reconstruction (Figure 1). The chest wall reconstruction method detailed by Fokin et al. was utilized, using the DePuy Synthes Matrix rib and sternal plating systems (DePuy Synthes, West Chester, PA) [8]. For rib fixation, video-assisted thoracoscopic surgery (VATS) was used to identify and verify the areas of chest wall weakness before making the chest wall incision and placing fixation plates and screws. All wounds were closed primarily (Figure 2). The 3M KCI-Acelity (San Antonio, TX) PREVENA™ ciNPT dressing was placed over each large incision at the end of the case, but not over thoracoscopy trocar incision sites (Figure 3). This ciNPT device was not removed until post-operative day 7 per manufacture's recommended guidelines (Figure 4). Each patient’s wounds were monitored while they were hospitalized and at regular intervals post discharge, for a period of two months, for evidence of SSI or dehiscence.

Data collection

Demographic data, such as age, gender, comorbidities, and mechanism of injury, were collected. Injury- and procedure-specific data, such as the number and location of fractures, the number of fixations, the procedure time, the wound classification score, and the location of each ciNPT, were recorded. Outcome data, such as the occurrence and date of superficial or deep SSI, length of intubation, mechanical ventilation, total and post-operative hospital length of stay, need for readmission or reoperation, and mortality, were recorded. Data were interpreted by counts, percentages, mean values, standard deviations, and p-values from two-sample t-testing where appropriate. A p-value <0.05 was determined to be significant.

## Results

Patient demographics

In our cohort, 12 patients (16.9%) required only sternal fixation, 49 patients (69%) required only rib fixation, and 10 patients (14%) required both. On average, 4.3 rib fixation plates were placed per patient (SD 2.4, range 0-17 fracture levels) to achieve sufficient stabilization. The mean age of this cohort was 63.3 years of age (range 23-90 years). A total of 66% of patients (47/71) were male, had a mean body weight of 84 kg, a mean height of 173 cm, and a mean body mass index of 28 kg/m^2^ (Table 1). The majority of patients suffered from motor vehicle accidents (31/71, 44%), falls (21/71, 30%), and motorcycle accidents (9/71, 13%). Seven percent (5/71) of patients were pedestrians struck by a car, 4% (3/71) suffered gunshot injuries, and 3% (2/71) experienced some form of crush injury not previously mentioned. A minority of patients (5/71, 7%) had a past history of surgery to their chest or chest wall. Comorbidities were present in 46% (33/71) of patients, including diabetes in 10% (7/71), hypertension in 35% (25/71), and some form of malignant neoplasm in 7% (5/71) of patients. Tobacco (13/71, 18%) and alcohol use (9/71, 13%) were more prevalent than illicit drug use (1/71, 1%).

**Table 1:** Patient demographic information including mechanism of injury and comorbidities BMI is calculated by dividing weight in kilograms by meters squared.

n = 71	No. or mean	% or SD
Age (years)	63.3	16.6
Female patients	24	34%
Weight (kg)	84.3	23.0
Height (cm)	173	10
BMI (kg/m^2^)	28.1	6.8
Prior chest surgery	5	7%
Mechanism of injury		
Motor vehicle accident	31	44%
Fall	21	30%
Other crush injury	2	3%
Car versus pedestrian	5	7%
Motorcycle accident	9	13%
Gunshot wound	3	4%
Comorbidities		
Congestive heart failure	1	1%
Diabetes mellitus type II	7	10%
Hypertension	25	35%
Hyperlipidemia	16	23%
Any cancer	5	7%
Renal or liver disorder	0	0%
Alcohol abuse	9	13%
Drug abuse	1	1%
Tobacco use	13	18%

Operative and wound care results

Using the Centers for Disease Control and Prevention (CDC) surgical wound classification system at the time of operation, 61% (43/71) of patients were classified as class I (clean) surgical procedures (Table 2) [7]. Due to the existence of other nearby infections or wounds, 18% (13/71) of patients were class II (clean-contaminated), and 17% (12/71) class III (contaminated). Only 4% (3/71) of patients were considered to have dirty or infected wounds (class IV). The average procedure length was 240 minutes (SD = 101 minutes, range 56-453 minutes) and this included the time for induction of and emergence from anesthesia. An average of 4.3 [median of 4] ribs were fixated per procedure (SD = 1.9). No difference was noted in the number of ribs fractured on the left (4.7) versus the right side (4.2, p = 0.31), nor the average number of ribs fixated on either side (4.3 left vs. 4.0 right, p = 0.53).

**Table 2:** Wound classification, sternal and rib fractures, and fixation results CDC, Centers for Disease Control and Prevention. Results are for wound classification, average operative time, number of ribs and sternum fractured, and the number of ribs and sternum fixed.

n = 71	No. or mean	% or SD
CDC surgical wound classification		
I	43	61%
II	13	18%
III	12	17%
IV	3	4%
Average surgery time (minutes)	240	101
Patients with sternal fracture and fixation	22	31%
Patients with rib fracture and fixation	59	83%
Patients with right rib fractures	31	44%
Mean number of right rib fractures	4.2	1.8
Mean number of right ribs fixated	4.0	1.8
Patients with left rib fractures	30	42%
Mean number of left ribs fractured	4.7	2.0
Mean number of left ribs fixated	4.3	1.9
Mean total rib fractures	4.7	2.5
Mean total ribs fixated	4.3	2.4

Outcomes of interest

There were 15 (21%) incidents of infection following chest wall reconstruction with ciNPT placement (Table 3). None of these cases were chest wall SSI. Six (8%) patients experienced pneumonia that resolved with antibiotics and conservative management. A total of 18 (25%) of the 71 patients remained intubated or on mechanical ventilation following chest wall reconstruction for an average of 7.6 days after surgery (SD = 3.6, range 1-15 days). Four (6%) patients who underwent chest wall reconstruction ultimately required placement of a tracheostomy to assist with ventilator weaning. There was one death in the study cohort; this death was attributed to the extent of multi-system trauma, the development of acute respiratory distress syndrome, as well as the presence of multiple comorbidities. Two-thirds of patients were discharged home following surgery (46/71, 65%), on average, on post-operative day 9.0 (SD = 6.8, range 1-30). Three (4%) patients were discharged on post-operative day 1, and 24 (34%) were discharged to sub-acute care facilities (7/71, 10%) or to skilled nursing facilities for rehabilitation (17/71, 24%).

**Table 3:** Hospital and post-operative course SSI, surgical site infection; VATS = video-assisted thoracoscopic surgery. Presented are the outcomes and post-operative course of our patient population, including infections during index admission, airway management, discharge data, and risk of readmission and reoperation.

n = 71	No. or mean	% or SD
Infection during admission	15	21%
Pneumonia during admission	6	8%
SSI during admission	0	0%
Patients requiring post-procedural intubation	18	25%
Mean number of days intubated	7.6	3.6
Patients with tracheostomy	4	6%
Total length of stay (days)	16.7	14.5
Post-operative length of stay (days)	11.8	11.1
Discharge home	46	65%
Discharge to skilled nursing facility	17	24%
Discharge to long-term acute care	7	10%
Deaths during the index admission	1	1%
Readmission related to fixation	2	3%
Fixation reoperation (hardware removal)	1	1%
Infection to VATS trocar sites	1	1%

Wounds were assessed by skilled nursing staff at day 7 (removal of dressing) and daily thereafter, at discharge, and periodically at clinic follow-up. There were only two incidences of infection after discharge that required readmission (3% of all patients). One patient experienced a VATS port site infection four weeks after the index procedure at an incision site that was not covered with the closed-incision negative pressure wound therapy. The other patient developed cellulitis along the sternal incision eight months after fixation. This was felt to be related to pressure necrosis of the skin from a weighted pendant the patient wore at all times, and ultimately necessitated removal of the sternal fixation hardware, wound debridement, and antibiotic therapy.

## Discussion

The results of this study demonstrate that after three years of practice and use, ciNPT can be effective in preventing SSI in trauma patients who undergo chest wall reconstruction. No patient in our study developed any complications attributable to the use of ciNPT. Other studies of ciNPT following orthopedic surgical procedures (extremities and spine), breast reconstruction, and cardiac procedures have shown a similar result [9-12]. Hyldig et al. in a 10-study meta-analysis were able to calculate a decreased pooled risk of SSI in patients who were treated with ciNPT versus standard wound care following surgery (5% vs. 9%) [9]. Their analysis also found that ciNPT prevents seroma formation and dehiscence and that for every 25 surgical cases using ciNPT, one incidence of SSI was prevented [9].

The prevention of SSI may be even more important in trauma patients with rib and sternal fractures, as procedures for chest wall reconstruction and stabilization are becoming more popular due to their efficacy in reducing the need for opioid pain medication, decreasing the duration of ventilation, and relieving sternal dehiscence and acute or chronic non-union of rib fractures [3,4,13-15]. Our study indicates that the use of ciNPT may also prevent SSI in patients with class III contaminated or class IV dirty wounds. Class III and IV wounds should undergo thorough high-pressure irrigation and debridement of any devitalized tissue before the placement of any fixation hardware. The incidence of SSI following sternal fixation has been shown to be as high as 7% and varied from 2% to up to 20% for patients after rib fixation [3]. Hardware infections can occur as distant as three and a half years after placement [3,16]. In our study, the only local infection occurred eight months after sternal fixation.

This study did not specifically evaluate the cost effectiveness of ciNPT in preventing SSI after chest wall reconstruction. Each PREVENA™ wound management kit costs approximately $500 and providers may charge $40 to $100 for application of the negative pressure therapy dressing [17]. While the dressing is in place, there are usually minimal ongoing additional costs. It is estimated that each SSI can cost up to $21,000 per admission and cause additional expenses with outpatient follow-up, as well as associated patient morbidity [18]. According to the meta-analysis by Hyldig et al., treating 25 patients to prevent one SSI would equal a cost of approximately $15,000, yielding a cost savings of up to $6,000 per SSI prevented [9].

Study limitations

A limitation of this study is the lack of a matched control group of patients who did not receive ciNPT and the retrospective nature of the data analysis. While we only encountered one patient with delayed infection, it cannot be ascertained whether any other patients developed a delayed infection beyond the two-month initial follow-up period. Lastly, this study would have benefited from a detailed cost analysis to determine the true cost effectiveness of using ciNPT for chest wall reconstruction.

## Conclusions

Today, ciNPT is well established in the management of open wounds in trauma and other surgical patients. Wound care management with ciNPT after rib fixation and/or chest wall reconstruction in trauma patients may be highly effective in preventing SSI and improving wound quality, thereby reducing morbidity and overall health care cost.
